# Investigation and Possibilities of Reuse of Carbon Dioxide Absorbent Used in Anesthesiology

**DOI:** 10.3390/ma13215052

**Published:** 2020-11-09

**Authors:** Bartłomiej Rogalewicz, Agnieszka Czylkowska, Piotr Anielak, Paweł Samulkiewicz

**Affiliations:** Institute of General and Ecological Chemistry, Faculty of Chemistry, Lodz University of Technology, Zeromskiego 116, 90-924 Lodz, Poland; agnieszka.czylkowska@p.lodz.pl (A.C.); piotr.anielak@p.lodz.pl (P.A.); psamulkiewicz@swspiz.pl (P.S.)

**Keywords:** soda lime, carbon dioxide, anesthesiology, absorbent, thermogravimetric analysis, PXRD analysis

## Abstract

Absorbents used in closed and semi-closed circuit environments play a key role in preventing carbon dioxide poisoning. Here we present an analysis of one of the most common carbon dioxide absorbents—soda lime. In the first step, we analyzed the composition of fresh and used samples. For this purpose, volumetric and photometric analyses were introduced. Thermal properties and decomposition patterns were also studied using thermogravimetric and X-ray powder diffraction (PXRD) analyses. We also investigated the kinetics of carbon dioxide absorption under conditions imitating a closed-circuit environment.

## 1. Introduction

Soda lime is one of the most popular carbon dioxide absorbents used in order to maintain a safe level of this gas. Its composition has slightly changed over time; however, calcium hydroxide is still the main component. Often an indicator signalizing its consumption is added, as well as small amounts of sodium (or potassium) hydroxide since NaOH is more reactive than Ca(OH)_2_. Moreover, the hygroscopic properties of NaOH reduce interphase mass transfer barriers and speed up the CO_2_ sorption process. Soda lime is most commonly used in environments characterized by reduced or no connection with fresh air, like anesthetic and diving apparatus or spacecraft. Such environments are commonly called “closed” and “semi-closed” circuit environments. It is an important issue since humans are there to the most degree, exposed to increased levels of CO_2_. Some of the most important examples of such systems, where soda lime and similar absorbents are used are anesthetic and diving apparatus, submarines, spacecraft and mine refuge chambers [1,2]. Breathing in such environments results in increased carbon dioxide concentrations. This may cause occurrence of several symptoms or even lead to death. In order to prevent it, absorbents based on alkali hydroxides are used to capture carbon dioxide, with soda lime being the most common one. Despite relatively low costs of production and simple operating principle, soda lime also has numerous flaws and limitations. It may undergo reaction with some of the gases used in general anesthesia, especially with sevoflurane, desflurane, isoflurane and enflurane to form a number of degradation products [3,4,5]. One of the gases, fluoromethyl 2,2-difluoro-1-(trifluoromethyl)vinyl ether, often abbreviated in the literature as *Compound A*, has been proven nephrotoxic to rats [6,7,8]. It may also contain viruses derived from the exhaled air, including rhinovirus, respiratory syncytial virus, parainfluenza virus, adenovirus, coronavirus, human metapneumovirus and influenza virus [9,10,11,12]. Under certain conditions, soda lime may also support the formation of carbon monoxide, one of the most toxic gases. Some of the factors that increase the possibility of carbon monoxide formation are the use of volatile anesthetic agents in question, their concentration and flow rate, dryness, type of used absorbent and the temperature in which absorption takes place [13,14,15,16]. Another complication is the need to control the absorbent exhaustion level since an excess of CO_2_ can cause hypercapnia. In order to signalize soda lime consumption, indicators like ethyl violet or ethyl orange are added. It does not, however, provide the necessary level of safety since sometimes these indicators may return to their previous color despite absorbent exhaustion [17]. Thus, capnometers are used in order to control the level of carbon dioxide in a patient’s organism. These devices are, however, relatively expensive and are not used in less developed areas. Soda lime with additions of indicators has also been withdrawn from U.S. Navy Fleet since it was suspected of releasing harmful compounds [18]. During the absorption process, extensive amounts of heat are produced, especially when baralyme (modification of soda lime in which calcium hydroxide is replaced with barium hydroxide) is used. Indicator color change monitoring and extensive heat emission additionally complicate soda lime use in diving apparatus. Furthermore, soda lime dust inhalation was observed, which may contribute to the occurrence of airway diseases in divers [19]. There are also no reports of reliable recycling methods of exhausted soda lime, which is most commonly considered medical waste. Taking into consideration all the mentioned problems and limitations, soda lime requires rigorous and careful handling. It is also the basis for seeking new alternative absorbents that would be more reliable and versatile. Some of them are carbonaceous materials, solid and liquid organic amines, mixtures of metals peroxides, hyperoxides or superoxides with water, membranes and zeolites [20]. There is no doubt that carbon dioxide plays one of the most significant roles taking into consideration both biological and environmental issues.

In this paper, we present a critical evaluation of soda lime performance as carbon dioxide absorbent. We have also investigated composition (volumetric analysis, photometric analysis) and thermal properties (thermogravimetric analysis, X-ray powder diffraction analysis) of two soda lime commercial samples, as well as proposed its possible recycling method.

## 2. Experimental

### 2.1. Materials and Analysis

All chemicals used during analysis were purchased from Avantor Performance Materials, Gliwice, Poland S.A.: pure calcium hydroxide, pure calcium carbonate, hydrochloric acid (1 mol·L^−1^), sodium hydroxide (4 mol·L^−1^), EDTA (0.05 mol·L^−1^), phenolphthalein, methyl orange, Patton and Reeder’s indicator. Analyzed soda lime samples came from the company producing carbon dioxide absorbents. The first sample was a fresh, unused sample, while the second one was used and considered exhausted prior to the research. The samples were marked as follows:

SL (F)—fresh soda lime sample;
SL (U)—used soda lime sample.

Chemical composition and thermal decomposition curves of samples were investigated using volumetric, photometric, thermogravimetric analysis. In order to better understand thermal decomposition pathways and to exclude the theoretical presence of other products of decomposition, a PXRD analysis was performed for sinters of samples SL (F) and SL (U) prepared at 950 °C. The sinters were obtained by heating the samples to the temperature defined from the thermal curves.

In the second part, we conducted an experiment under conditions imitating carbon dioxide absorption in closed circuit anesthetic apparatus, which allowed us to draw conclusions about the kinetics of carbon dioxide absorption and soda lime performance as a carbon dioxide absorbent. Chemical composition and thermal destruction ways after absorption were investigated in the same way as in the first part of our study. These samples were marked in the following way:

SL (5 min)—fresh soda lime sample after 5 min of carbon dioxide absorption;
SL (15 min)—fresh soda lime sample after 15 min of carbon dioxide absorption.
SL (30 min)—fresh soda lime sample after 30 min of carbon dioxide absorption.

**Figure 1:**
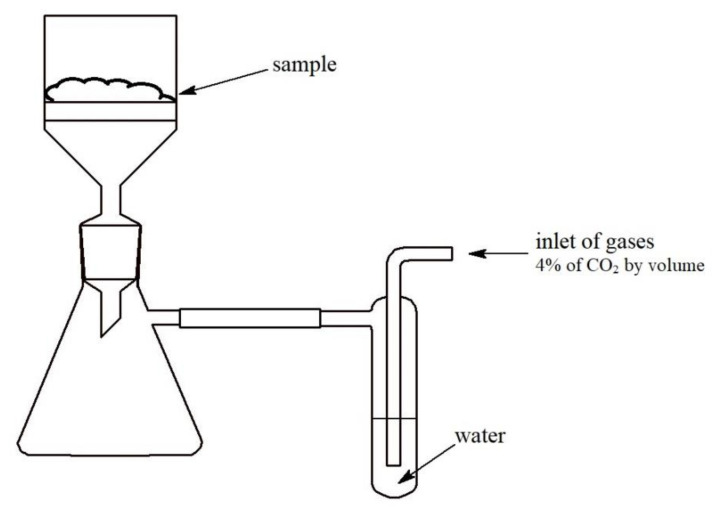
Experimental setup for carbon dioxide absorption under conditions imitating closed-circuit environment.

The experimental setup is schematically shown in Figure 1. A compressor was used to flow atmospheric air through a water bubbler and then a packed bed of sorbent. The carbon dioxide concentration in the inlet air (C_o_) was around 4% (average concentration of carbon dioxide in exhaled air). The experiment was conducted at room temperature (23–25 °C), and relative humidity was maintained at around 55% during the experiment.

### 2.2. Methods and Instruments

A bigger batch of each sample was ground in a mortar. After homogenization, around 1.0 grams of each sample was stirred in 1 L of distilled water on a magnetic stirrer for 24 h. Suspensions prepared this way were then investigated using volumetric analysis. In order to ensure repeatability of results, no less than three portions per each sample were collected and titrated. Powders resulting from grinding in a mortar were investigated using thermogravimetric analysis. We have also performed thermal decompositions of two main soda lime components—calcium hydroxide Ca(OH)_2_ and calcium carbonate CaCO_3_. Volumetric analysis was performed using automatic burettes at room temperature (23–25 °C). *P* alkalinity and *M* alkalinity determinations were performed using 2 mol·L^−1^ hydrochloric acid solution in the presence of phenolphthalein and methyl orange, respectively. Calcium ion concentration determinations were performed using 0.05 mol·L^−1^ EDTA solution in the presence of Patton and Reeder’s indicator. Photometric analysis was performed using BWB-XP flame photometer (BWB Technologies, Newbury, England). The content of sodium in samples was measured at an analytical spectral line 589 nm with the limit of detection 0.02 ppm. Thermal behavior and decomposition patterns of samples were investigated using IRIS 209 (Netzsch, Selb, Germany) in the temperature range 25–980 °C at a heating rate of 4°·min^−1^ in flowing dynamic nitrogen atmosphere (v = 30 mL·min^−1^) using platinum crucibles; as reference material, platinum crucibles were used. PXRD analysis was performed using a X’Pert Pro MPD diffractometer (PANalytical, Malvern, England) in the Bragg–Brentano reflection geometry using CuK_α_ radiation in the 2θ range 5–90° with a step of 0.0167° and exposure per step of 50 s.

## 3. Results and Discussion

The performed analyses allowed us to determine the composition of the investigated samples SL (F), SL (U), SL (5 min), SL (15 min), SL (30 min). Volumetric and thermogravimetric analyses allowed us to calculate the percentage of the contents of calcium hydroxide and calcium carbonate for each sample. The contents of water were derived from thermal decomposition, as dehydration is the first process of thermolysis. Photometric analysis allowed us to establish the content of sodium hydroxide in investigated samples. Table 1 presents the collected data.

Determination of each component’s content was done separately using different analytical techniques, which may have caused propagation of error. This is the reason why, in some samples, the summed contents of components may exceed 100%. The biggest error occurred in SL (15 min) sample (contents sum up to 102.32%); however, it was still within the error tolerance.

### 3.1. Thermal Decomposition of Samples: SL (F), SL (U), Samples After Absorption: SL (5 min), SL (15 min) and SL (30 min)

**Figure 2:**
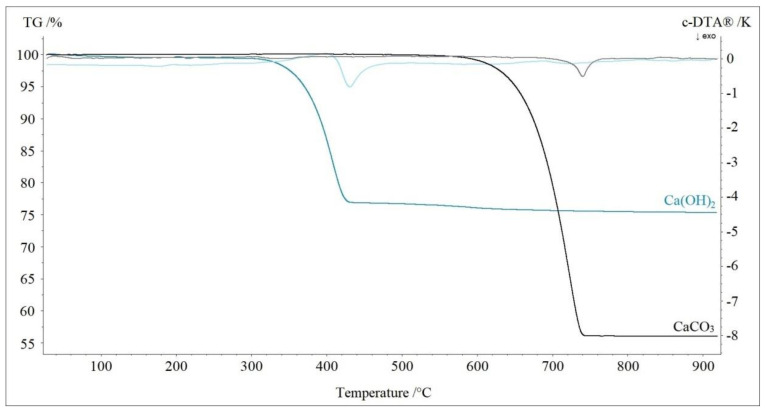
TG-DTA curves of decomposition of calcium hydroxide and calcium carbonate in nitrogen.

Figure 2 presents the thermal decomposition of calcium hydroxide and calcium carbonate. Thermolysis began at 280 °C for calcium hydroxide (DTA peak at 430 °C) and at 560 °C for calcium carbonate (DTA peak at 740 °C). Mass losses and decomposition curves were consistent with reactions that took place during the process. For calcium hydroxide, it was the release of one molecule of water, and for calcium carbonate, it was the release of a molecule of carbon dioxide. The final product of decomposition in both cases was pure calcium oxide.

**Figure 3:**
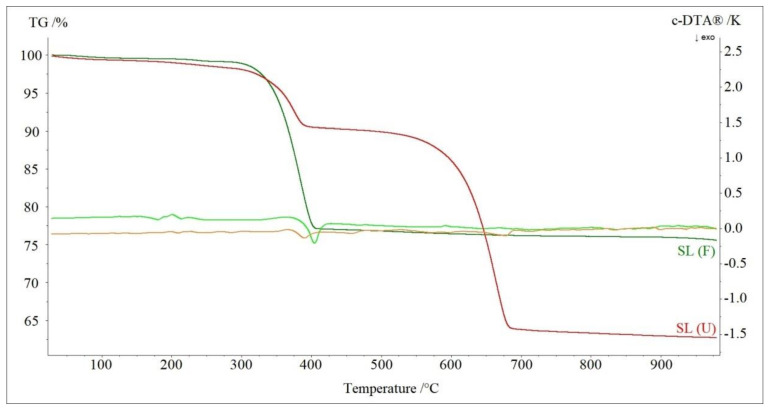
TG-DTA curves of decomposition of fresh soda lime sample (SL (F)) and used soda lime sample (SL (U)) in nitrogen.

Figure 3 presents TG-DTA curves of the investigated fresh soda lime sample SL (F) and the used sample SL (U) that came from a hospital and were considered exhausted. It is clear that the first step of decomposition was the dehydration of samples. For SL (F), the sample mass loss related to this process was 0.89% at a temperature range 25–275 °C, while for the SL (U) sample, it was 1.93% at a temperature range 25–300 °C. In the second step, one of the samples’ components decomposed—calcium hydroxide. For the SL (F) sample, it took place above 275 °C (DTA peak at 405 °C), while for the SL (U) sample—at a temperature range of 300–400 °C (DTA peak at 390 °C). It was clearly visible that above 400 °C, for the SL (U) sample, decomposition of calcium carbonate took place (DTA peak at 675 °C).

**Figure 4:**
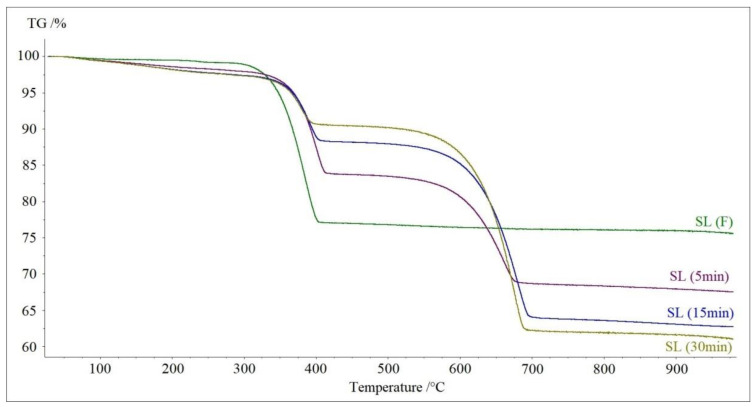
TG curves of decomposition of SL (F) sample and samples after carbon dioxide absorption: SL (5 min), SL (15 min) and SL (30 min) in nitrogen.

Figure 4 presents the TG curves of investigated soda lime samples after 5, 15 and 30 min of carbon dioxide absorption, as well as the TG curve of the SL (F) sample (after 0 min of absorption). The decomposition path was analogous in all cases. The first step (up to 300 °C) was associated with dehydration. It is clear that along with the increasing time of CO_2_ absorption, the content of water increased. We could also observe how the second step of decomposition, associated with decomposition of calcium hydroxide, shortened, while the last step, associated with decomposition of calcium carbonate, increased along with time. These curves also show that the process of absorption slowed down with time.

**Figure 5:**
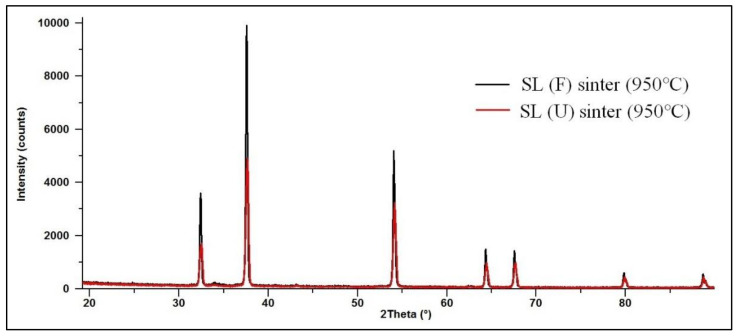
X-ray powder diffraction patterns of analyzed SL (F) and SL (U) sinters prepared at 950 °C.

Soda lime is a mixture of different chemicals, and thus its thermal decomposition is a multistage process. In all cases, the first step is dehydration. Later, decomposition of calcium hydroxide and calcium carbonate takes place. In order to thoroughly investigate the thermal properties of such absorbents, we decided to study the composition of two sinters prepared at the end of the decomposition process (950 °C). Both the fresh sample’s (SL (F)) and the used sample’s (SL (U)) sinters were prepared. Their X-ray powder diffraction patterns are shown in Figure 5. These patterns correlate very well with calcium oxide, proving it is a final product of the thermal decomposition.

### 3.2. Chemical Kinetics of Carbon Dioxide Absorption by SL (U) Sample

Three samples of SL (F) were exposed to CO_2_ absorption for 5 min, 15 min and 30 min, and then analyzed in the same way as SL (F) and SL (U) samples. The absorption of carbon dioxide and water is a multistage process. The first stage involves the formation of carbonic acid from CO_2_ and water. Then, NaOH (or KOH) added in small amounts acts as an activator to speed up the process through the formation of sodium (or potassium) carbonate. It can also be concluded that the absorption and the hydration of CO_2_ and the formation of CO_3_^2−^ are rapid steps, and the dissolution of Ca(OH)_2_ is the slowest step of the carbonation process [21,22]. Calcium hydroxide reacts with the carbonates within minutes to form an insoluble precipitate of calcium carbonate as well as results in a regeneration of NaOH [23]. Some carbon dioxide may also react directly with Ca(OH)_2_ to form calcium carbonates, but this reaction is much slower. In addition, calcium bicarbonate may be formed on the surface of the sorbent particles. The higher solubility of bicarbonate enhances CO_2_ diffusion through the bulk of the particle [24]. Soda lime is exhausted when all hydroxides become carbonates.

**Figure 6:**
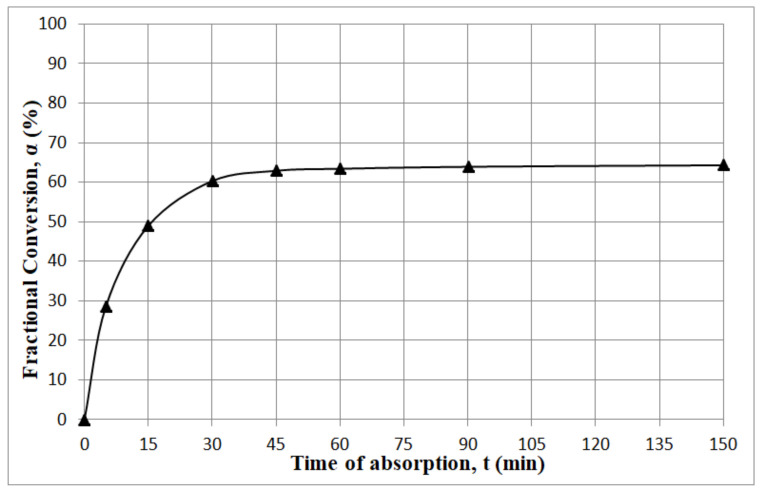
Relationship between the fractional conversion of soda lime and time of carbon dioxide absorption.

Figure 6 shows the experimental results of CO_2_ sorption on soda lime as a relationship between conversion rate *α* and time *t*. It clearly indicates that the conversion of the sorbent was incomplete and would be difficult to reach under the typical working conditions. According to the results shown in the figure, about 20–30 min from the beginning of the experiment, the carbonation rate slowed down noticeably. We can observe that the curve is composed of two sections. The initial upslope of the curve depicts the fastest rate of carbonation; its initial rate was 3.3 min^−1^. After 30 min, the reaction slowed down and reached a rate of 0.17 min^−1^. It was a result of significant limitations of CO_2_ transport from the surface to the bulk of the sorbent particles, and differentiation between kinetics-controlled and diffusion-controlled ranges occurs.

The reaction rate of a solid-state process, $\frac{d\alpha }{dt}$, can be related to the process temperature, *T*, and to the fraction reacted, *α*, by means of the following general equation [25]:

(1) $$\frac{d\alpha }{dt}=k\cdot f\left(\alpha \right)$$

where *k* is a constant rate.

The kinetic curve of CO_2_ absorption of soda lime can be described by the pseudo-first or pseudo-second order kinetic equation [26,27]. In the first section of the kinetic curve, the carbonation is controlled by the surface reaction, whereas in the second section, a heterogeneous system is controlled mainly by diffusion [28]. Assuming a driving force of CO_2_ removal to be proportional to the difference between its concentrations in sorbent at any time prior to equilibrium and its concentration at equilibrium, we can use the equation:

(2) $$\frac{d\alpha }{dt}=k_{n}{\left(\alpha_{e}-\alpha \right)}^{n}$$

**Figure 7:**
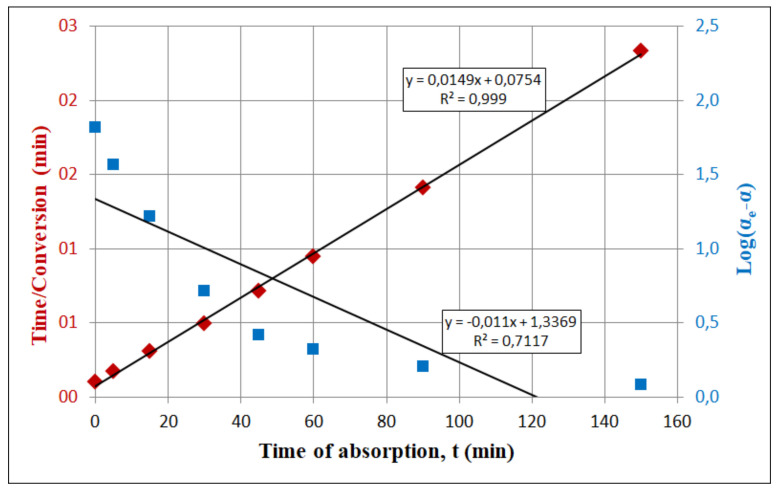
Linearized equation of the pseudo-first (right axis) and pseudo-second (left axis) order kinetics models.

The fittings of the experimental data to the linear form of the two kinetic models, i.e., pseudo-first order and pseudo-second order, are shown in Figure 7.

The values of the correlation coefficient for linear forms of both kinetic equations are significantly different. The pseudo-second order model describes the kinetic data better than pseudo-first order model when the process is diffusion-limited [29]. The obtained values of the correlation coefficients were, therefore, 0.999 and 0.712, respectively. Thus, pseudo-first order model does not cover both stages of the CO_2_ sorption, i.e., the chemical reaction and the diffusion process. However, the carbonation rate constant determined using first order reaction was greater than for the second order reaction and amounted to 0.011 min^−1^ and 0.0022 min^−1^, respectively. Experimental data have shown that the carbonation process ends before all lime is converted into a calcium carbonate [23]. On the other hand, the first, fast absorption stage is completed within one hour, and the experimental and calculated values of fractional conversion (Figure 6) were in good agreement with values calculated for both kinetic models: 65.5% and 67.1%, respectively [27].

**Figure 8:**
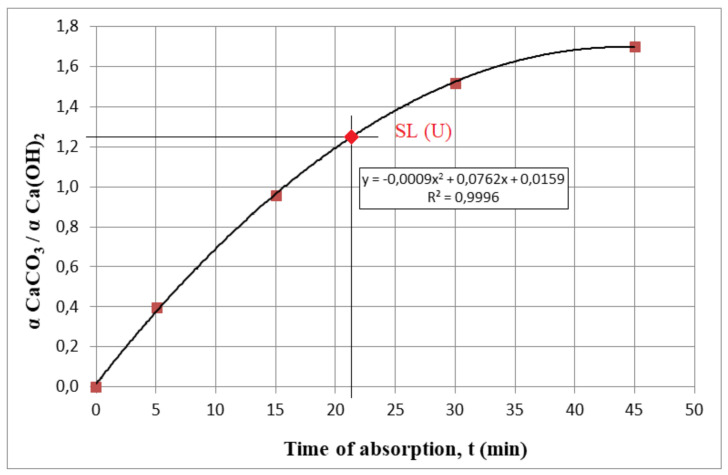
Relationship between calcium carbonate amount and calcium hydroxide amount ratio and time.

One of the possible ways to express soda lime exhaustion rate is a relationship between calcium carbonate content and calcium hydroxide content $\frac{\alpha \;CaCO_{3}}{\alpha \;Ca{\left(OH\right)}_{2}}$ in the bed and time *t*. This relationship is presented in Figure 8.

We used this relationship to determine what time of absorption corresponds with the chemical composition of the SL (U) sample. For example, a bed exhaustion rate of 1.247 could be obtained after 21.7 min.

## 4. Conclusions

Using various analytical techniques, we determined the chemical composition of several soda lime samples: fresh sample, exhausted sample after use in hospital and three samples after carbon dioxide absorption under conditions imitating semi-closed circuit apparatus. Thermogravimetric and XRD analyses comprehensively described the thermal properties and decomposition ways of the investigated samples. This product decomposed in a stable manner, releasing water and carbon dioxide. It is possible to recycle and reuse soda lime in different forms; however, the calcination process would require relatively high temperatures. On the other hand, high temperatures would ensure the biological neutrality of recycled soda lime. Calcium oxide itself could be reused in many different areas, e.g., in absorption and desiccation, in the construction industry or in the manufacturing of chemicals.

Soda lime is a fairly efficient carbon dioxide absorbent that has been used for a long time. It has, however, some limitations and drawbacks that require further investigations, as it is a product used in environments where dependability is a factor of great importance. One of the issues that should be addressed is possible interactions between absorbent and anesthetic gases, which can lead to the release of harmful compounds. Another limitation is the speed of carbon dioxide absorption, which is the highest at the beginning of the process and slows down relatively fast. On the surface of the soda lime granules, water forms a film less than three molecular layers thick, and the reaction rate is reduced [21]. As the carbonation proceeds, the product particles precipitate on the surface of Ca(OH)_2_ and cover it a thicker, porous deposit layer, which inhibits the exchange of reacting species between the surface of calcium hydroxide and bulk solution. Therefore, the diffusion rate of reacting species is an important factor affecting the final stage of carbonation. The carbonation of Ca(OH)_2_ was observed to stop before one hour. However, carbonation may go on by diffusion through the covering layer, but its rate is too slow to be detected in the range of carbonation time used [28]. Thorough research on soda lime properties is an important step in the evaluation of its performance as an agent responsible for preventing carbon dioxide poisoning. Lack of data concerning the kinetics of this process causes this problem to be still very interesting and important in both anesthetic and medical science. Such data would provide a reliable tool to compare different types of absorbents and thus would allow proper absorbent choice taking into consideration all other aspects of the environment or apparatus. Our research, only to some extent, covers the main problems of soda lime use. Additional further studies must be performed in order to ensure the required level of safety and efficiency and to determine the best recycling method.

## Figures and Tables

**Table 1:** Composition of investigated samples.

Sample	Percentage Content [% (m/m)]			
	Ca(OH)_2_	CaCO_3_	H_2_O	NaOH
SL (F)	96.78	0	0.89	2.50
SL (U)	35.85	60.50	1.93	2.10
SL (5 min)	63.23	34.09	2.08	2.28
SL (15 min)	42.34	55.09	2.74	2.15
SL (30 min)	31.41	64.45	2.95	2.04
